# SARS-CoV-2 spike protein expression drives post-acute coagulopathy

**DOI:** 10.1128/jvi.01255-25

**Published:** 2026-01-21

**Authors:** Chih-Feng Tien, En-Ju Lin, Wei-Hsiang Tsai, Wan-Ting Tsai, Ming-Yu Chen, Yu-Siang Su, Han-Chieh Wu, Yueh-Tzu Chiu, Wan-Ju Tung, Yi-Ping Kuo, Yu-Wen Su, Hsin-Wei Chen, Feng-Jui Chen, Tsung-Hsien Chuang, Hsiang-Tsui Wang, Guann-Yi Yu

**Affiliations:** 1 National Institute of Infectious Diseases and Vaccinology, National Health Research Institutes 50115, Zhunan, Taiwan; 2 Immunology Research Center, National Health Research Institutes 50115, Zhunan, Taiwan; 3 Graduate Institute of Biomedical Sciences, China Medical University 38019, Taichung, Taiwan; 4 Graduate Institute of Medicine, College of Medicine, Kaohsiung Medical University 38023, Kaohsiung, Taiwan; 5 Department of Pharmacology, School of Medicine, National Yang Ming Chiao Tung University 390197 , Taipei, Taiwan; Loyola University Chicago - Health Sciences Campus, Maywood, Illinois, USA

**Keywords:** SARS-CoV-2, spike, Delta, Omicron, S1 subunit, microcoagulation, VSV, organ dysfunction, IGFBP-1, CXCL13, fibrinogen, long COVID, PASC

## Abstract

During the COVID-19 pandemic, multiple SARS-CoV-2 variants emerged, each with distinct pathogenicity and transmissibility. This study investigated the role of the viral spike (S) protein in disease progression, focusing on the highly virulent S variant. The Delta S protein exhibited enhanced cleavage efficiency at the S1/S2 junction, resulting in partial dissociation of the S1 subunit, with detectable levels of extracellular S1. Unexpectedly, transient expression of Ancestral and Delta S protein induced by a recombinant vesicular stomatitis viral vector caused mild pulmonary inflammation, neutrophil activation, microthrombosis, and ~40% mortality in transgenic K18-hACE2 mice between 8 and 16 days, similar to post-COVID sequelae. The diseased mice displayed splenic atrophy and systemic inflammation, with elevated serum IGFBP-1 and CXCL13 levels. Consistent with the animal findings, serum samples from long COVID patients showed significantly elevated IGFBP-1 levels. CXCL13 levels were particularly elevated in patients with more severe long COVID symptoms. Notably, treatment with the antiplatelet agent aspirin significantly reduced both mortality and weight loss in mice exposed to Delta S protein expression. These findings suggest that SARS-CoV-2 S protein-associated coagulation and systemic inflammation during infection may contribute to the development of post-acute sequelae of COVID-19.

**IMPORTANCE:**

Our study investigates the distinctive pathogenic properties of the SARS-CoV-2 spike (S) protein from highly virulent variants, with a particular focus on its delayed pathological effects in mice. Using a vesicular stomatitis virus (VSV) vector to transiently express the Ancestral and Delta variant S proteins in K18-hACE2 mice, we observed minimal acute symptoms initially; however, approximately 40% of the mice developed mild pulmonary inflammation, neutrophil activation, and microthrombosis, leading to death between 8 and 16 days post-infection. This delayed pathology was accompanied by elevated circulating levels of CXCL13 and IGFBP-1. Consistent with these findings, serum samples from long COVID patients also showed significantly increased IGFBP-1 levels, while CXCL13 levels were particularly elevated in individuals with more severe long COVID symptoms. These findings provide important observational evidence that may guide future mechanistic studies on long COVID and inform the development of potential therapeutic approaches.

## INTRODUCTION

Severe acute respiratory syndrome coronavirus 2 (SARS-CoV-2) emerged from Wuhan, China, at the end of 2019 and caused coronavirus disease (COVID-19). With a high transmission rate, SARS-CoV-2 infection became a global pandemic, leading to the evolution of many variants from Ancestral SARS-CoV-2 strain. Variants of concern have changes that affect virulence, transmissibility, vaccine effectiveness, or diagnostic testing, such as Alpha, Beta, Gamma, Delta, and Omicron variants (1–3). For example, Delta variant infection in humans leads to severe clinical symptoms, particularly in the lower respiratory tract. Compared to Ancestral and Alpha strains, Delta variant infection increases hospitalization risk, prolongs the duration of hospital admission, and heightens the need for emergency care in COVID-19 patients (4–7). In contrast, Omicron variant infection mainly affects the upper respiratory tract and leads to mild symptoms. With a short incubation period of 2–3 days and a high transmission rate (R0 value of 10), the Omicron variant has evolved into over 300 sub-lineages (8–10).

Beyond these acute clinical features, growing evidence highlights the long-term health consequences of SARS-CoV-2 infection, collectively referred to as long COVID, also known as post-acute sequelae of COVID-19 (PASC) (11). According to the World Health Organization, PASC is defined as the persistence or emergence of new symptoms approximately three months after the onset of COVID-19, lasting for at least two months and not explained by an alternative diagnosis. The predominant clinical manifestations of PASC involve multiple organ systems and include brain fog, dyspnea, memory impairment, insomnia, and various mental health and cognitive disorders. Full vaccination with multiple doses of COVID-19 vaccines substantially reduces the risk of developing PASC (12). Numerous studies have shown that PASC can develop following infection with any SARS-CoV-2 variant, although the risk appears lower after Omicron infection compared to Delta infection (13–16). The pathogenesis of PASC is multifactorial, involving several interrelated mechanisms, such as viral persistence, microthrombosis, autoimmune responses, and chronic inflammation (17). Despite these findings, the precise mechanisms underlying PASC remain incompletely understood. In particular, how acute pathophysiological events induced by SARS-CoV-2 infection contribute to long-term sequelae is still unclear.

To elucidate the biological basis of these clinical observations, comparative studies in animal models have revealed distinct pathogenic profiles among SARS-CoV-2 variants. In mice, infection with the Delta variant induces more severe lung injury and higher mortality than infection with the Omicron variant (18–20). The genetic mutations of SARS-CoV-2 variants are primarily in the spike (S) protein, which is the structural protein on the viral envelope that interacts with angiotensin-converting enzyme 2 (ACE2) molecule on the cell surface for infection (21). Newly synthesized S protein undergoes posttranslational modifications in the endoplasmic reticulum (ER) and Golgi apparatus, including glycosylation, palmitoylation, and protease cleavage (22). The S protein is cleaved into S1 and S2 subunits by furin protease in the trans-Golgi network. The mutations and cleavage efficiency of the S protein in different variants may influence virus transmissibility, cell-to-cell fusion, and other functions (23–26).

SARS-CoV-2 infection causes pulmonary disease and extrapulmonary tissue damage, further leading to oxidative stress, endothelial cell dysfunction, and thrombosis (27–29). Damaged endothelial cells trigger coagulation and thrombosis in blood vessels. Neutrophils are recruited to the damage site and release neutrophil extracellular traps (NETs) through a process called NETosis. These NETs contain histones, nucleosomes, tissue factors, and myeloperoxidase (MPO), which further enhance thrombosis (30). The expression levels of coagulation factors, such as vWF, factor VIII, and soluble P-selectin, and fibrinogen degradation product (D-dimer and fibrin) in serum are highly associated with severe COVID-19 patients (31–34). It has been shown that S protein can downregulate the expression of junctional protein and increase endothelial barrier permeability (35–37). The S protein can also enhance neutrophil recruitment and NETosis upon endothelial damage (35, 38, 39). The receptor-binding domain (RBD) of S protein interacts with spleen tyrosine kinase (Syk)-coupled C-type lectin member 2 (CLEC2) on platelets, thereby activating platelets to promote NET formation and thromboinflammation (40). Moreover, SARS-CoV-2 can activate Syk-coupled C-type lectin member 5A (CLEC5A) and Toll-like receptor 2 (TLR2) in the platelet, leading to the release of extracellular vesicles that further induce NETosis (41). As endothelial dysfunction and NETosis could lead to multi-organ injury in COVID-19 patients (42–44), the role of S protein in SARS-CoV-2 pathogenesis requires further investigation.

To understand the association between the S protein and COVID-19 disease severity, we aim to characterize the S protein of SARS-CoV-2 variants in protein cleavage efficiency, virus packaging efficiency, and *in vivo* pathogenicity. Our results suggest that the Delta S protein is effectively cleaved, and the S1 subunit is easily to be secreted to extracellular space. Surprisingly, transient expression of Ancestral and Delta S protein in mice leads to mild inflammation, neutrophil activation, and microcoagulation in the lung and mouse death. The study links S protein to post-acute coagulation.

## MATERIALS AND METHODS

### Cell culture and reagents

BHK21 c15 cells were cultured in MEM alpha medium (Cytiva, Marlborough, MA) containing 10% FBS (Cytiva), 2 mM L-glutamine solution (Biological Industries, Israel), 1× Penicillin Streptomycin (PS) solution (100 IU/mL Penicillin and 100 μg/mL Streptomycin, Corning, New York), and 1× HEPES buffer (Gibco, ThermoFisher). Human ACE2 (hACE2) overexpression in both 293T and BHK21 cells was kindly provided by Dr. Chia-Yi Yu from the National Institute of Infectious Diseases and Vaccinology, NHRI, Taiwan. 293T or 293T/ACE2 cells were maintained in DMEM/High glucose medium (Cytiva) supplemented with 1× PS solution and 10% FBS. BHK21/ACE2 cells were maintained in RPMI medium (Cytiva) with 1× PS solution and 5% FBS. Antibodies used for immunoblotting were anti-SARS-CoV-2 S2 (Genetex, Hsinchu, Taiwan; GTX632604), anti-SARS-CoV-2 S2 (cell signaling, Danvers, MA; cat#84534), anti-SARS-CoV-2 RBD (S1, homemade) (45), anti-SARS-CoV-2 S1 (cell signaling; cat#56996), anti-VSV-M (Absolute, Boston, MA), anti-tubulin (Sigma, St. Louis, MO), and anti-beta-actin (Genetex).

### Recombinant vesicular stomatitis virus (rVSV)-spike variants and virus precipitation

The human codon-optimized genes of Delta and Omicron spike variants lacking C-terminal 19 amino acid (SΔ19), with or without additional R682G and S813Y mutations, were inserted into VSVΔG-GFP-2.6 plasmid (Kerafast, Boston, MA). The recovery of rVSV-SΔ19 virus from plasmid DNA and generation of replication-competent viruses (Rep) followed methods described previously (45–48). The VSVΔG/G control virus was generated from the VSV empty vector and enveloped with exogenous VSV-G protein (45). The titer determination of rVSV-SΔ19 or Rep viruses was performed by counting GFP-positive cells after serial dilution or by TCID50 in BHK21-ACE2 cells. For virus concentration, rVSV-containing culture supernatant was mixed with Polyethylene Glycol (PEG) solution (Sigma; cat#MAK343) and incubated overnight at 4°C. The virus was then precipitated by centrifugation at 4,000 rpm for 30 min, and the precipitate was resuspended in virus re-suspension buffer (Sigma).

### Mice

K18-human ACE2 transgenic mice were obtained from the Jackson Laboratory and bred at NHRI. Mice aged 3–8 months were intranasally infected with rVSV viruses at 1 × 10⁶ pfu/mouse, 2 × 10**^7^** pfu/mouse, or 1 × 10⁸ pfu/mouse. Aspirin treatment (Sigma-Aldrich, cat# A2093) was administered to mice via oral gavage at a dose of 10 mg/kg per mouse starting 24 h post-infection and continued once daily for 17 consecutive days. Mouse body weight and survival were monitored daily. Mice showing symptoms such as more than 10% weight loss and reduced vitality were classified as sick; otherwise, they were considered recovered.

### Human serum

Serum samples and associated metadata from long COVID patients (*N* = 80) and control individuals (*N* = 15) were obtained from the National Health Research Institutes Biobank (TSPN No. 24044) under approved IRB protocol (EC1130901-E). The non-COVID-19 control group included individuals diagnosed with hepatic adenoma, focal nodular hyperplasia, or hemangioma. Based on symptom severity assessed by physicians through clinical evaluations and standardized inventories, the long COVID group was stratified into four subgroups: mild (*N* = 69), moderate (*N* = 6), severe (*N* = 3), and critical (*N* = 2).

### Immunofluorescence staining

Frozen lung tissue sections were fixed in 10% neutral buffered formalin solution (Sigma; cat#HT501128) and subjected to immunostaining with myeloperoxidase (MPO) (R&D Systems, Minneapolis, MN, USA; cat#AF3667) or F4/80 (Invitrogen; Thermo Fisher) antibodies. The tissue sections were counterstained with DAPI contained in mounting medium (BIOTIUM; cat#23002) and examined using a fluorescent microscope (Olympus iX73).

### Immunohistochemistry

Paraffin-embedded spleen sections were rehydrated and subjected to antigen retrieval using Universal HIER Antigen Retrieval Reagent (ab208572, Abcam, Cambridge, UK), according to the manufacturer’s instructions. An anti-fibrinogen antibody (Dako; Agilent) was used to detect fibrinogen deposition.

### Flow cytometry

The mouse splenocytes were collected in FACS buffer (2% BSA, 2mM EDTA, 0.01% NaN_3_ in PBS) and treated with 1× RBC lysis buffer solution (eBioscience; ThermoFisher). The splenocytes were stained with antibodies against CCR2, CD19, Ly6G, CD11b, CD4, CD11c, CD8, CD3, F4/80, Ly6C, B220, and CD45 (Biolegend, San Diego, CA) and analyzed by Flow Cytometry (BD FACScalibur).

### Enzyme-linked immunosorbent assay (ELISA)

Specific protein expression levels in mouse serum or plasma were quantified using the Mouse IGFBP-1 DuoSet ELISA (DY1588), Mouse EGF DuoSet ELISA (DY2028), Mouse CXCL13/BLC/BCA-1 ELISA Kit (MCX130), Mouse CXCL13/BLC/BCA-1 DuoSet ELISA (DY470), Human IGFBP-1 ELISA (DY871), and Human CXCL13/BLC/BCA-1 ELISA (DY801) (R&D Systems, Minneapolis, MN, USA), according to the manufacturer’s instructions. The anti-S specific IgG titer in mouse serum was assessed using the SARS-CoV-2 S2 extracellular domain recombinant protein (S2ECD, SinoBiologicals, Beijing, China) as the antigen, as previously described (45).

### Cytokine array

Cytokine detection in the mouse plasma and lung sample was performed using the Mouse XL Cytokine Array Kit (R&D Systems, Minneapolis, MN, USA; ARY028), following the manufacturer’s instructions. The detected expression signals were quantified using VisionWorks 9.1 software (Analytik Jena). Changes in cytokine expression were calculated as follows: ((average expression signal of a specific cytokine in sample plasma) − (average expression signal of the same cytokine in control plasma)) / (average expression signal of the same cytokine in control plasma).

## RESULTS

### Dissociation of S1 subunit during spike (S) protein expression

**Fig 1:**
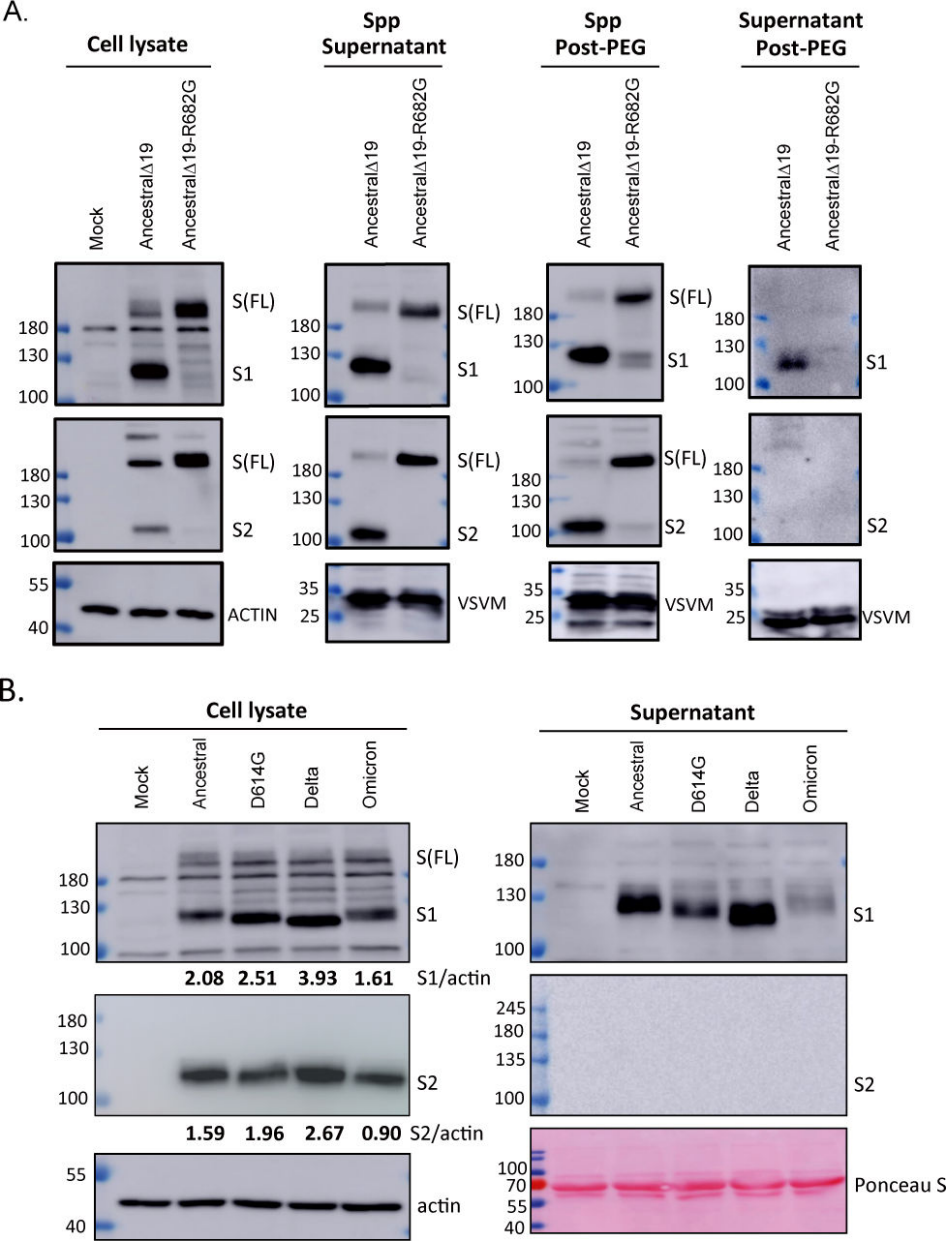
SARS-CoV-2 spike (S) variants release free S1 subunit. (**A**) 293T cells were transfected with plasmid encoding Ancestral S wild-type (WT) or R682G mutant for 24 h. The transfected cells were then infected with the VSVΔG/G virus (m.o.i = 3) to produce S-pseudotyped virus particles (Spp) for 24 h. Spp in culture medium was further precipitated with polyethylene glycol (PEG). The transfected cell lysate (without virus infection) and Spp were subjected to immunoblotting. (**B**) 293T cells were transfected with plasmid DNA carrying S variants to introduce S expression for 36 h, and S protein level in cell lysate and supernatant was examined by immunoblotting.

The S1 subunit of the SARS-CoV-2 S protein has functions that include activating immune cells, increasing endothelial permeability, and contributing to blood clotting (36, 49, 50). The potential for the S1 subunit to remain in complex with the S2 subunit or be secreted as a free form was investigated. A vesicular stomatitis virus (VSV)-based vector (VSVΔG-GFP) was used to generate S-pseudotyped virus particles (Spp). To enhance Spp packaging, a plasmid encoding an S mutant (Ancestral strain) with a deletion of the C-terminal 19 amino acids (SΔ19) was transfected into 293T cells, and cleavage of the S protein into S1 and S2 subunits was observed (Fig. 1A). Upon VSVΔG-GFP infection, pseudotyped virus particles containing S1 and S2 subunits were detected in the culture supernatant. The S1 and S2 subunits on the virus particles were precipitated with polyethylene glycol (PEG), but some S1 proteins remained in the post-PEG supernatant, indicating that these S1 proteins were not in complex with S2 and existed in a free form. Expression of the S protein with the R682G mutation at the S1/S2 junction in cells or Spp predominantly resulted in an uncleaved form, reducing the generation of free S1 (Fig. 1A). These findings suggest that free S1 can be generated during the formation of virus particles.

To assess whether S1 can be released directly from cells, 293T cells were transfected with plasmid DNA to express the full-length S protein from the Ancestral strain and its variants (D614G, Delta, and Omicron). The S, S1, and S2 proteins of the Ancestral strain and all variants were detected by immunoblotting in cell lysates (Fig. 1B, left panel). The Delta S variant exhibited the highest cleavage efficiency, while the Omicron S variant showed the lowest cleavage efficiency in 293T cells. A similar cleavage pattern was observed in A549 lung epithelial cells (Fig. S1). Interestingly, the S1 subunit was detected in the culture medium, particularly from cells expressing the Ancestral and Delta S proteins (Fig. 1B, right panel). The S2 subunit, which contains a transmembrane domain, was not detected in the culture medium. In contrast, cells expressing the Omicron S protein had the lowest levels of free S1 in the culture medium. These results suggest that S1 may dissociate from the S1/S2 complex and be secreted from S protein-expressing cells.

To determine whether the S1 protein can be actively secreted in the absence of the S2 subunit, plasmids encoding the S1 protein fused with an Fc tag (~26 kDa) were constructed. To prevent potential cleavage at the S1-Fc junction, the ^682^RRAR^685^ sequence of the S1 protein was modified to ^682^GSAS^685^ (51). All S1-Fc recombinant proteins of the Ancestral, Delta, and Omicron variants were expressed in 293T cells, but only the Delta S1-Fc fusion protein was actively secreted into the culture medium (Fig. S2). The Delta variant spike exhibited high cleavage efficiency and a high tendency for extracellular S1 subunit secretion. Previous studies have demonstrated that the SARS-CoV-2 S protein can induce NETosis in neutrophils (39–41). Both the Ancestral and Delta S pseudoparticles (Spp) triggered NETosis in neutrophils (Fig. S3A). Moreover, extracellular S1 subunits released into the culture supernatant from 293T cells expressing the Ancestral or Delta S protein also triggered NETosis (Fig. S3B). These results indicate that the dissociated S1 subunit retains functional activity capable of activating neutrophils.

### Replication-competent recombinant VSV-SΔ19 of Delta and Omicron variants

The SARS-CoV-2 S protein containing an R682G mutation at the furin cleavage site, or an additional S813Y mutation at the TMPRSS2 cleavage site, decelerates S protein processing and enables the recombinant VSV-S (rVSV-S Ancestral) virus to become replication-competent (45). Since the Omicron S protein exhibited slow cleavage efficiency and a stable S1/S2 complex (Fig. 1B), the rVSV-S (Omicron) might more easily become replication-competent compared to other variants. To test this hypothesis, the SΔ19 genes of Delta and Omicron were inserted into a VSVΔG-GFP vector to generate rVSV-S. The R682G and R682G+S813Y mutations were also introduced into the SΔ19 gene to enhance rVSV-S virus replication in some designs (Fig. S4A).

**Fig 2:**
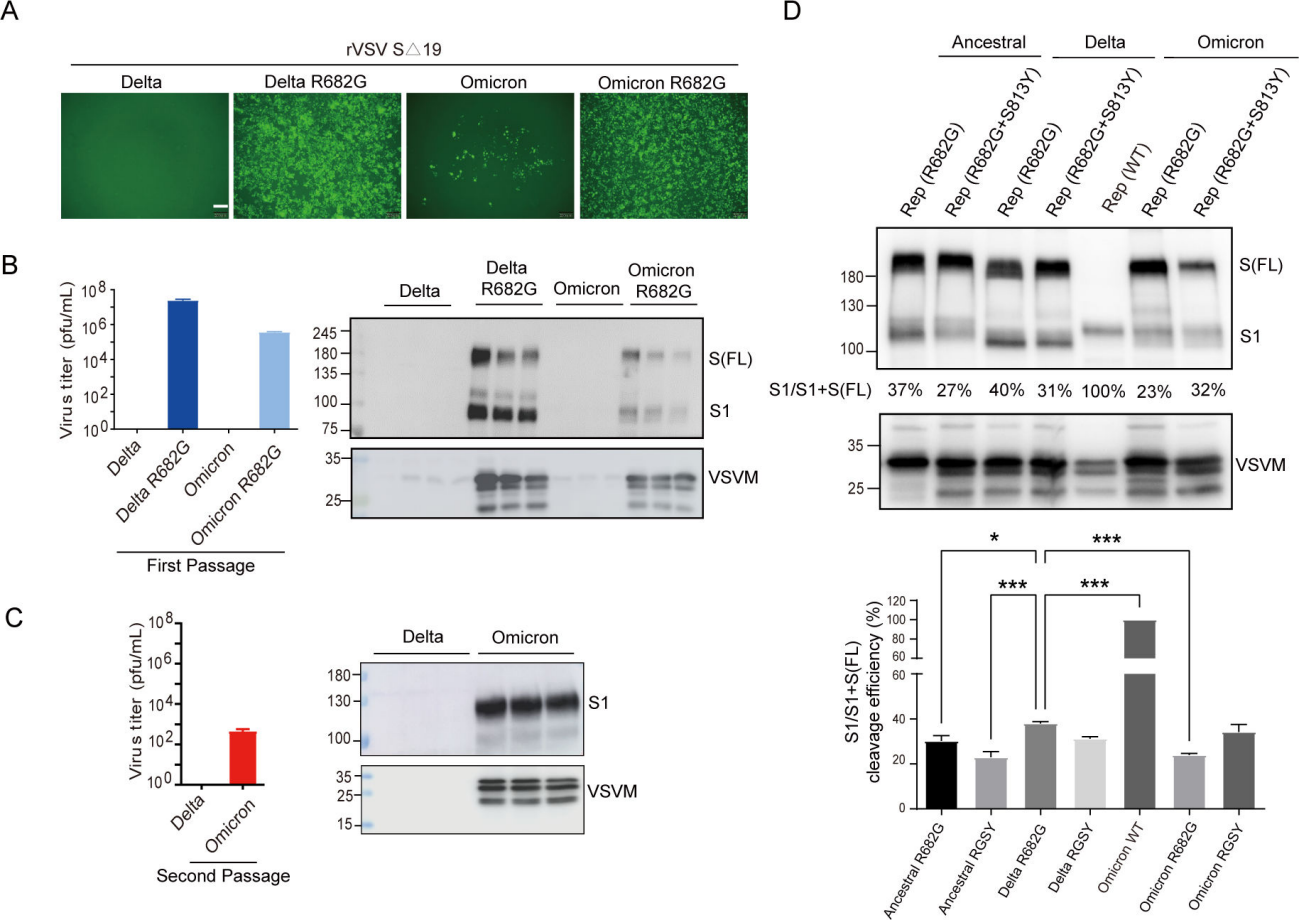
Generation of replication-competent recombinant VSV expressing Delta and Omicron S protein. (**A**) The Delta and Omicron S genes (wild-type or R682G mutants) with C-terminal 19 amino acid truncation were inserted into VSVΔG-GFP-2.6 plasmid, and the recombinant viruses (rVSV-SΔ19) were rescued by co-transfected with helper plasmids into 293T cells. The rescued viruses were used to infect 293T cells, and virus replication was monitored with GFP expression. Scale bar: 200 μm. rVSV-SΔ19 virus titer and S protein expression on rVSV particles in culture supernatant at first (**B**) and second (**C**) passages were examined. (**D**) The S protein expression pattern of replication-competent rVSV-SΔ19 (Rep) of Ancestral, Delta, and Omicron variants with R682G or R682G+S813Y mutation was examined by immunoblotting. The cleavage efficiencies of S protein were quantified using VisionWorks. Error bars represent SEM and *N* = 3 (Dunnett’s test, *P < 0.05 and ***P < 0.001).

As shown in Fig. 2A and B, the first passage of rVSV-S(Delta) did not show any virus replication, as evaluated by GFP detection, virus titration, or immunoblotting. However, once the R682G mutation was introduced, the virus became replication-competent Rep-S(Delta-R682G). In contrast, replication of rVSV-S(Omicron) was observed through GFP expression after the first passage. Virus titer and viral protein of Rep-S(Omicron) virus were detected after the second passage (Fig. 2C), suggesting that the Omicron S1/S2 complex is indeed stable enough for rVSV packaging without the additional R682G mutation. When the S protein expression pattern of the Rep viruses was compared, the cleavage efficiency of the Rep-S(Delta R682G) and Rep-S(Ancestral R682G) virus remained higher than that of Rep-S(Omicron R682G) viruses (Fig. 2D). The addition of the S813Y mutation slightly reduced the cleavage efficiency of the S protein in the Rep-S(Delta R682G+S813Y) virus. The RNA genome sequences of these Rep viruses were further analyzed by nanopore sequencing (Fig. S4B). The Rep-S(Delta R682G) virus contained an additional V705M mutation, while the Rep-S(Omicron) virus harbored a D405N mutation. Moreover, comparison of the cleavage efficiency of S variants carrying the R682G mutation across different experimental systems revealed that the rVSV-S system exhibited substantially higher cleavage efficiency than both the pseudotyped virus system and the S protein DNA transfection system (Fig. 2D & Fig. S5). These results indicate that the elevated S1 levels observed are a characteristic specific to the Rep-S(R682G) system.

### Transient Ancestral and Delta S(R682G) protein expression leads to K18-hACE2 mouse death

**Fig 3:**
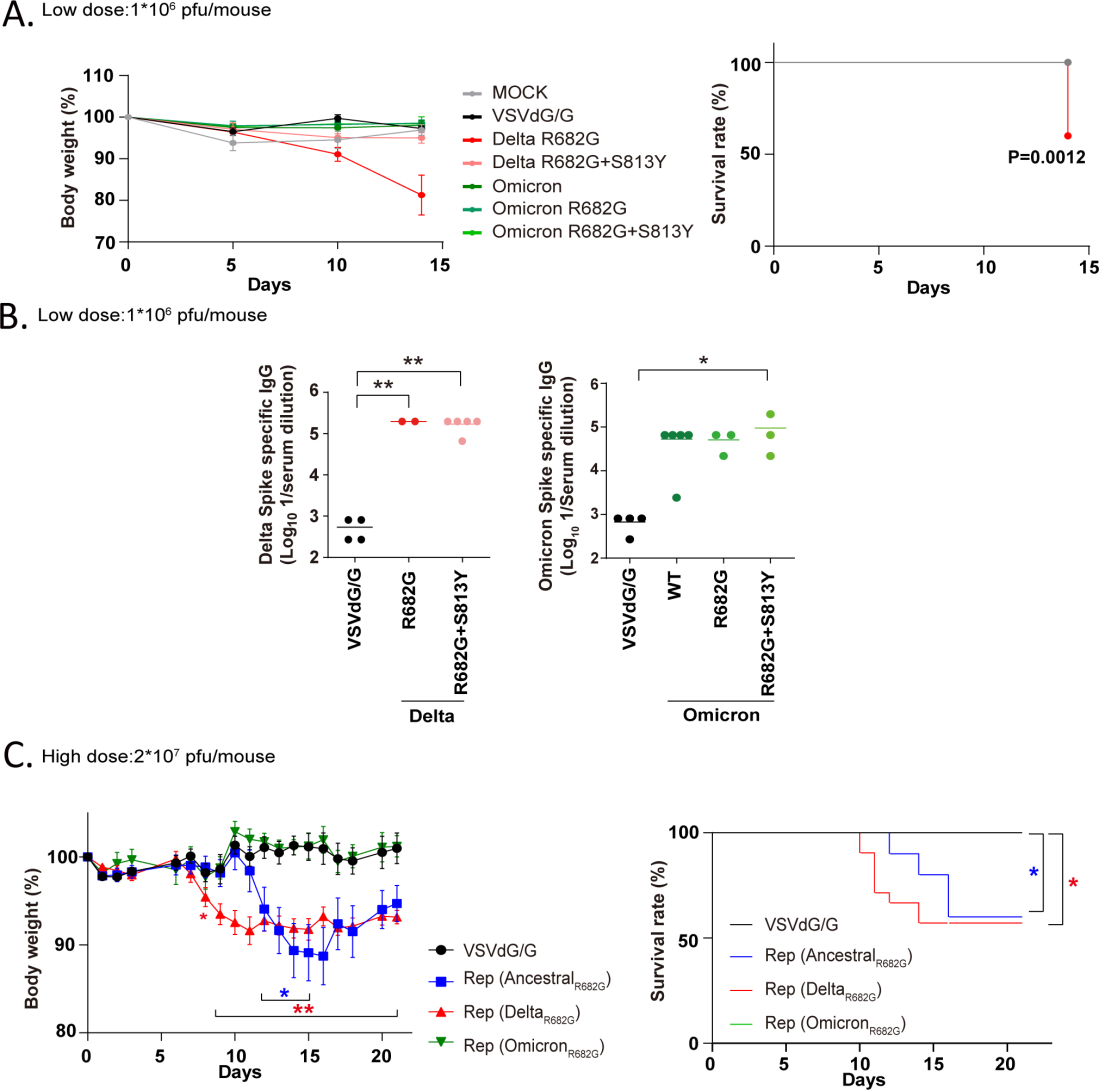
Ancestral and Delta Rep-S (R682G) virus infection induces death in hACE2 mice. (**A**) hACE2 mice were intranasally infected with VSVΔG/G (control virus) or Rep viruses (Delta and Omicron mutants) (low dose: 1 × 10^6^ pfu/mouse; mock *N* = 4; other groups *N* = 5), and body weight loss and survival rate were monitored for two weeks (log-rank test). (**B**) IgG antibody against specific spike antigen (Delta or Omicron spike protein) in mouse serum from 14 days post-infection (dpi) was evaluated by ELISA (one-way ANOVA, *P < 0.05 and **P < 0.01). (**C**) K18-hACE2 mice were intranasally infected with a high dose of Rep-S (R682G) virus (2 × 10⁷ pfu/mouse). Body weight loss and survival were monitored over a three-week period. Group sizes were VSVΔG/G (*n* = 9), Ancestral R682G (*n* = 10), Delta R682G (*n* = 21), and Omicron R682G (*n* = 10) (mixed-effects analysis and log-rank test, *P < 0.05 and **P < 0.01).

To investigate potential differences in the expression of Delta and Omicron S proteins in mice, Rep viruses were utilized to introduce transient expression of these proteins in K18-hACE2 mice (1×10^6^ pfu /mouse, administered intranasally). Since the VSV vector is sensitive to innate immunity, protein expression is known to decline within 24 h (45). Mice exposed to the control virus (VSVΔG/G), Rep-S(Omicron), Rep-S(Omicron R682G), Rep-S(Omicron R682G+S813Y), and Rep-S(Delta R682G+S813Y) did not exhibit any symptoms over the course of two weeks. Unexpectedly, three out of five mice infected with Rep-S(Delta R682G) experienced significant body weight loss (>20%), with two mice succumbing within 14 days (Fig. 3A). Specific antibodies against the Delta or Omicron S proteins were detected in the mice infected with Rep viruses, indicating that all Rep viruses effectively induced S protein expression in mice (Fig. 3B).

When hACE2 mice were intranasally inoculated with a high dose of the Rep-S(R682G) virus (2 × 10⁷ pfu/mouse), 43% of those infected with the Delta variant exhibited weight loss and succumbed between days 8 and 13 post-infection (Fig. 3C). Similarly, 40% of mice infected with the Rep-S(Ancestral R682G) virus showed body weight loss around days 11–16, with mortality occurring slightly later than in the Delta-infected group. To determine whether the deaths were due to ongoing virus replication or other infectious agents, nanopore sequencing was performed on lung tissue from the *sick* animals. No significant viral or bacterial activity was detected (data not shown), suggesting that the observed inflammation and coagulation may be driven by immune responses to the transient expression of the S R682G protein. These findings indicate that transient expression of the Ancestral and Delta S(R682G) proteins elicits a pathogenic response in mice.

### Pulmonary inflammation and thrombosis following transient expression of Ancestral and Delta S (R682G) protein

**Fig 4:**
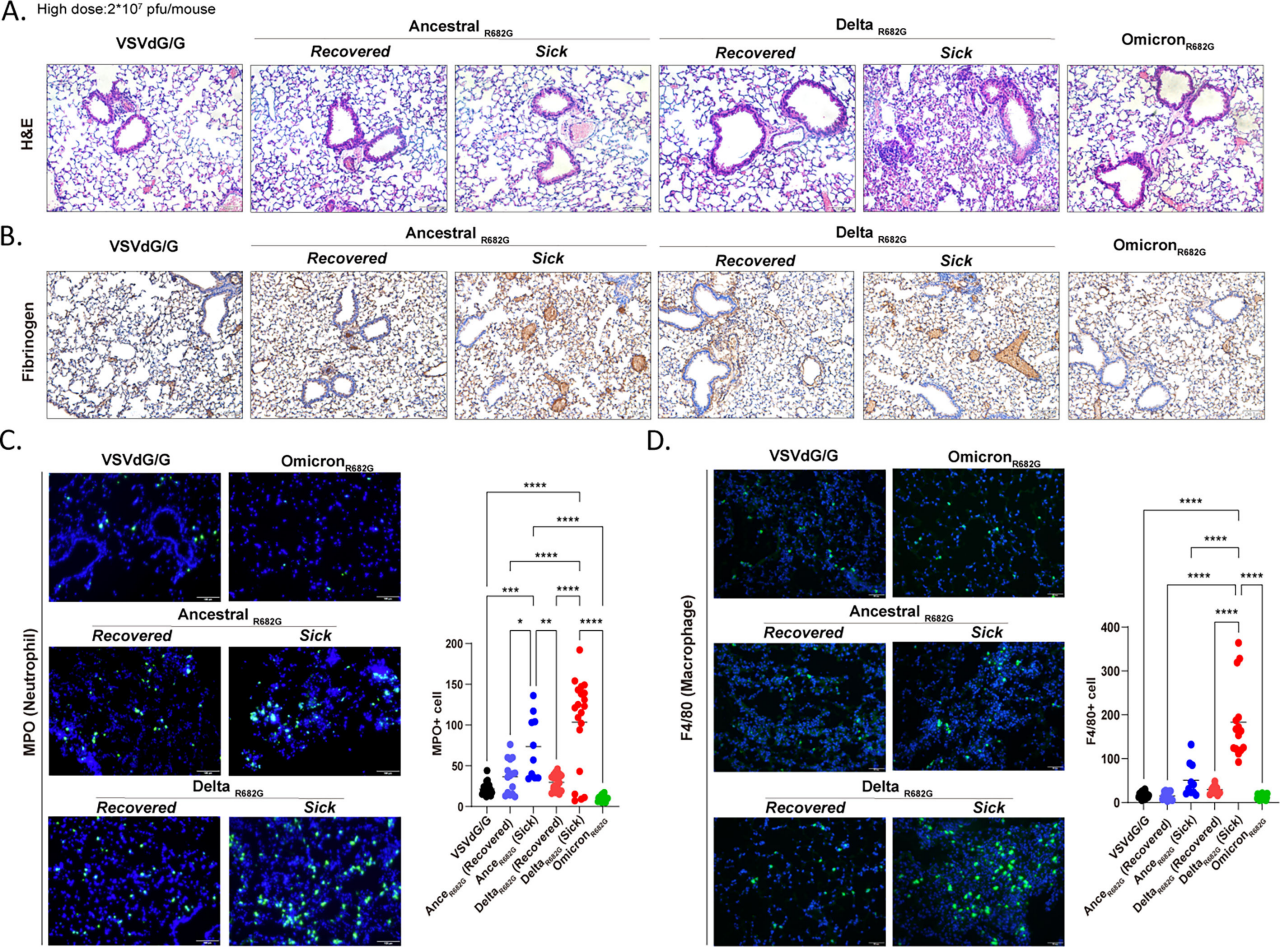
Transient Ancestral and Delta S protein expression induces microcoagulation and neutrophil recruitment in mouse lung. (**A–D**) Lungs from hACE2 mice infected with VSVΔG/G or Rep-S (R682G) viruses (high dose: 2 × 10⁷ pfu/mouse) were collected at 20 dpi or at the onset of severe symptoms (moribund stage). Lung sections from moribund or *recovered* mice were stained with hematoxylin and eosin (H&E) (**A**) and analyzed by immunohistochemistry using an anti-fibrinogen antibody (**B**). Scale bar: 100 μm. Neutrophils and macrophages in the lung sections were detected by immunofluorescence with anti-MPO (**C**) and anti-F4/80 (**D**) antibodies, respectively. Scale bar: 50 or 100 μm. For quantification, five random fields were imaged per sample, and the number of positively stained immune cells was counted in each field (one-way ANOVA, *P < 0.05, **P < 0.01, ***P < 0.001, and ****P < 0.0001).

Mice exposed to the Ancestral and Delta Rep-S (R682G) viruses were categorized based on clinical presentation: those exhibiting significant weight loss were designated as the *sick* group, while those without symptoms or weight loss were classified as the *recovered* group. To investigate the cause of mortality, lung tissues were examined by H&E staining and immunohistochemistry (Fig. 4). Lungs from the *sick* mice infected with Rep-S(Delta R682G) displayed reduced airspace, marked immune cell infiltration, and red blood cell aggregation (Fig. 4A). Fibrinogen staining revealed increased fibrinogen deposition within both the blood vessels and alveolar spaces of mice exposed to the Ancestral and Delta R682G variants, particularly in the *sick* groups (Fig. 4B), suggesting excessive coagulation activity. Infiltration of myeloperoxidase-positive (MPO^+^) neutrophils was observed in the lungs of *sick* animals (Fig. 4C), whereas macrophages (F4/80^+^) were evident only in the lungs of Rep-S(Delta R682G)–infected mice (Fig. 4D). These phenotypes were absent in the Omicron R682G group. To assess whether SARS-CoV-2 variants differentially induce coagulation activity, fibrinogen deposition was examined by immunohistochemistry in the lungs of hamsters infected with live SARS-CoV-2 viruses. Infections with the Ancestral and Delta variants resulted in greater fibrinogen deposition in the lungs compared to infection with the Omicron variant (Fig. S6).

Fibrinogen deposition was also detected in the livers of mice from the Rep-S(Delta R682G)–exposed *sick* group (Fig. S7), accompanied by elevated serum levels of aspartate aminotransferase (AST/GOT) and alanine aminotransferase (ALT/GPT) (Table S2). To assess systemic coagulation responses, mRNA expression levels of coagulation-related genes were analyzed across multiple tissues. In Rep-S(Delta R682G)–exposed mice, expression levels of F3 in the lung, F8 in the spleen, and SerpinE1 in the liver were correlated with body weight loss (Fig. S8). These findings suggest that an exaggerated coagulation response may be a key factor contributing to disease severity and mortality in mice exposed to the Rep-S(Delta R682G) virus.

### S protein-induced coagulation leads to splenic lymphocyte depletion

**Fig 5:**
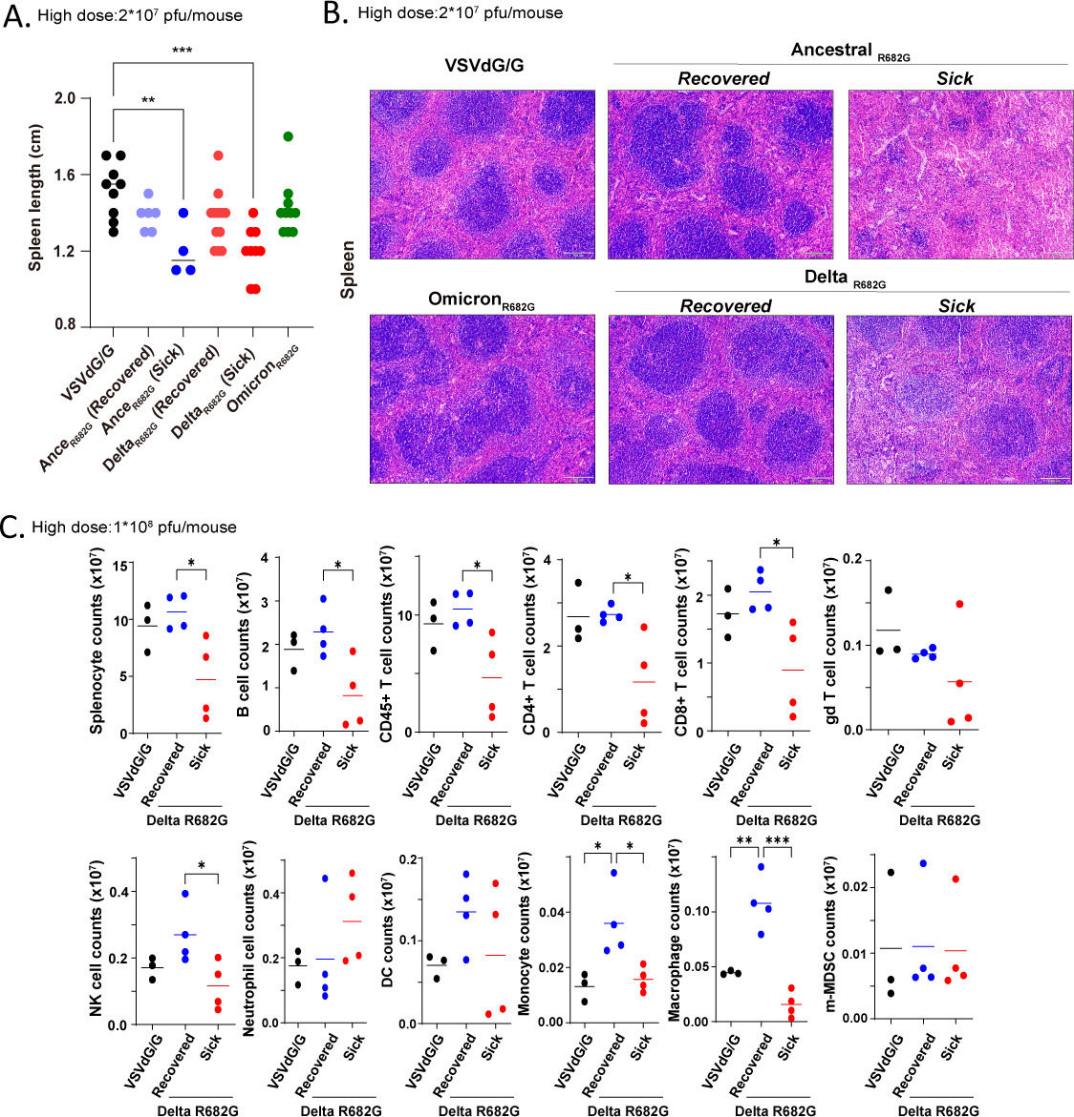
Transient S protein expression induces splenic atrophy. (**A–B**) Spleens from hACE2 mice infected with VSVΔG/G or Rep-S (R682G) viruses (high dose: 2 × 10⁷ pfu/mouse) were collected at 20 dpi or at the moribund stage. (**A**) Spleen lengths were measured (one-way ANOVA; *P* < 0.01, *P < 0.001). (**B**) Spleen sections were stained with hematoxylin and eosin (H&E). Scale bar: 100 μm. (**C**) To examine splenic immune cell populations, hACE2 mice were infected with VSVΔG/G or Rep-S (Delta R682G) viruses (high dose: 1 × 10⁸ pfu/mouse). Splenocytes collected at 12 dpi were analyzed by flow cytometry (one-way ANOVA, *P < 0.05, **P < 0.01, and ***P < 0.001).

Following exposure to Rep-S(R682G) viruses, spleens from the Ancestral and Delta *sick* groups were markedly smaller than those from the control group (Fig. 5A). Histological analysis revealed a reduction in white pulp in these atrophic spleens (Fig. 5B). Flow cytometry analysis of splenocytes revealed that these *sick* mice had a reduced total splenocyte count, along with decreased populations of B cells, CD4^+^ T cells, CD8^+^ T cells, and NK cells (Fig. 5C). In contrast, myeloid cell populations, such as monocytes and macrophages, were elevated in the spleens of *recovered* virus-exposed mice but remained low in the *sick* group. These findings indicate that most cell populations were compromised in these *sick* animals. Taken together, transient expression of Ancestral and Delta S protein induces splenic atrophy.

### IGFBP-1 and CXCL13 are potential biomarkers for S protein-induced coagulopathy

**Fig 6:**
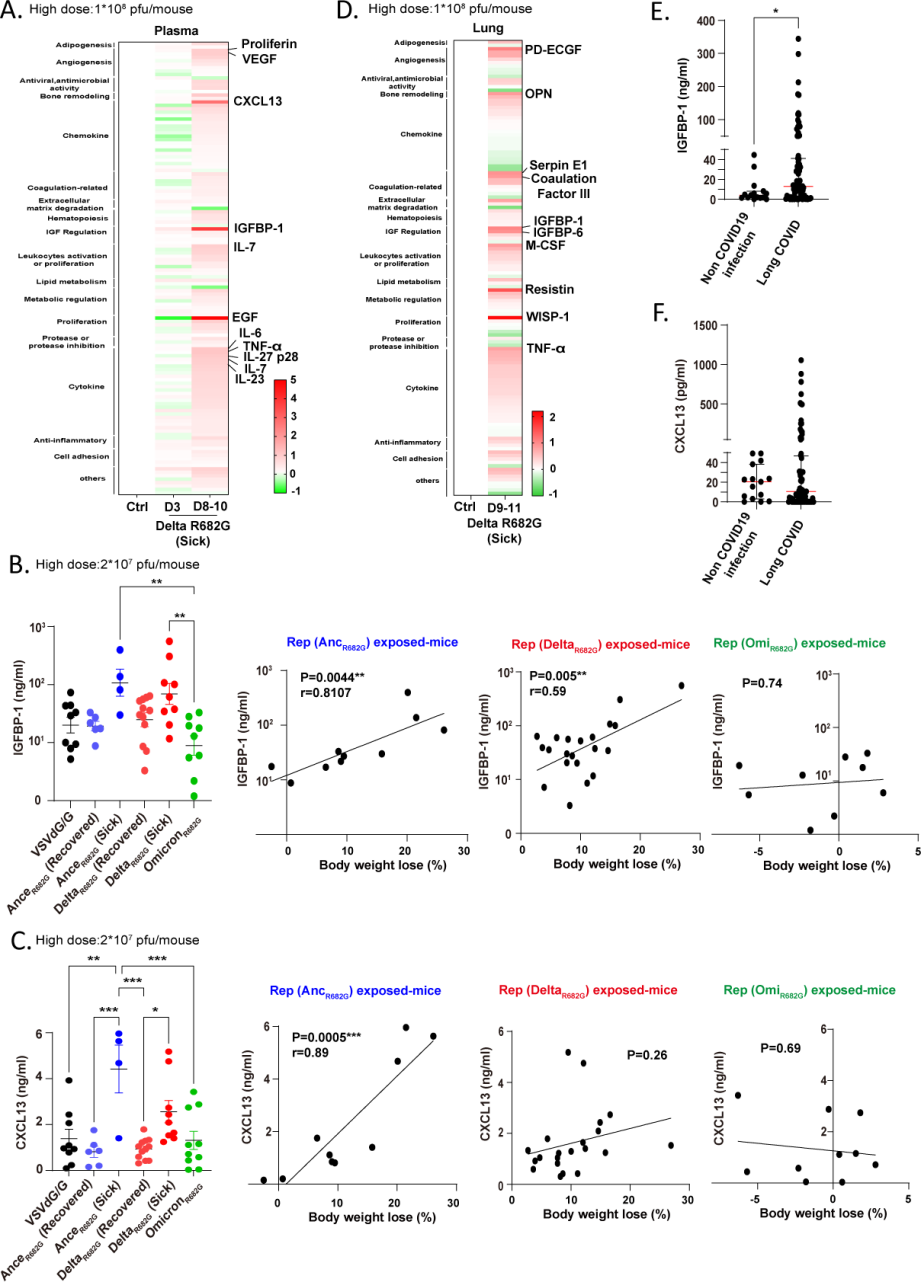
IGFBP-1 and CXCL13 are potential biomarkers for coagulopathy induced by the S protein and for identifying long COVID patients. (**A**) Cytokine expression in plasma from hACE2 mice infected with VSVdG/G or Rep-S Delta R682G viruses (high dose:1 × 10^8^ pfu/mouse) at 3 and 8–10 dpi was analyzed using a cytokine array. The changes in cytokine expression were calculated and presented in a heatmap, with the top 10 cytokines showing the greatest increase marked. (**B–C**) Plasma levels of CXCL13 and IGFBP-1 in infected mice (high dose: 2 × 10^7^ pfu/mouse) were measured by ELISA (one-way ANOVA and Pearson correlation, *P < 0.05, **P < 0.01, and ***P < 0.001). (**D**) Cytokine expression in lung tissue from hACE2 mice infected with VSVdG/G or Rep-S Delta R682G (1 × 10^8^ pfu/mouse) at 9–11 dpi was profiled by cytokine array. Detailed cytokine levels in plasma and lung samples are provided in the supplementary data. (**E–F**) Serum levels of IGFBP-1 and CXCL13 were measured by ELISA in individuals without prior COVID-19 infection (*n* = 15) and in long COVID patients (*n* = 80). The non-COVID-19 group included individuals diagnosed with hepatic adenoma, focal nodular hyperplasia, or hemangioma. (Mann–Whitney *U* test, *P < 0.05).

To identify a sensitive biomarker for lung inflammation and coagulation, plasma from Rep-S(Delta R682G) virus-exposed mice was analyzed using a Proteome Profiler cytokine array (Fig. 6A; Table S3). On day 3 post-exposure, only a few cytokines and chemokines showed slight elevation in the plasma of *sick* animals, including CCL6 (50% increase), insulin-like growth factor-binding protein-1 (IGFBP-1, 50% increase), LDL-R (50% increase), and pentraxin 2 (60% increase). At later time points (days 8–10), numerous inflammatory mediators were elevated. Notably, epidermal growth factor (EGF, 490% increase), IGFBP-1 (380% increase), and CXCL13 (280% increase) were significantly induced in Rep-S(Delta R682G) virus-exposed diseased mice. ELISA analysis revealed that plasma IGFBP-1 levels were positively correlated with body weight loss in both the Ancestral and Delta groups (Fig. 6B). A positive correlation for CXCL13 was observed only in the Ancestral group (Fig. 6C), while no significant correlation was found for EGF expression (Fig. S9). Moreover, cytokine levels in lung tissue from mice exposed to the Rep-S(Delta R682G) virus were also examined using a cytokine array (Fig. 6D; Table S3). IGFBP-1 showed a significant increase (110%) in diseased mice, consistent with observations in plasma samples. Coagulation factor III (F3) was also elevated, aligning with their RNA expression levels (Fig. S8A). These findings suggest that IGFBP-1 and CXCL13 could potentially serve as biomarkers for S protein-induced coagulopathy.

Transient S protein expression induced by Ancestral and Delta Rep-S(R682G) virus infection triggered delayed immunopathological symptoms, which may represent a potential mechanism contributing to long COVID (or PASC). To explore this, serum levels of CXCL13 and IGFBP-1 were evaluated in long COVID patients. Serum samples from long COVID patients and non-COVID-19 controls, obtained from the National Health Research Institutes Biobank, were subjected to CXCL13 and IGFBP-1 ELISA assays (Fig. 6E and F). While overall CXCL13 levels did not show a statistically significant difference between the control group and long COVID patients, elevated levels of CXCL13 were detected in a subset of long COVID cases (Fig. 6F). Further analysis revealed that high CXCL13 levels were significantly associated with patients who experienced moderate to critical long COVID, as well as those with comorbidities such as diabetes, chronic kidney disease, and hypertension (Table 1). In contrast, IGFBP-1 levels were significantly elevated in long COVID patients compared to non-COVID-19 controls (Fig. 6E). Additionally, long COVID patients over the age of 50 exhibited higher IGFBP-1 levels than those under 50. Patients with comorbidities also showed increased IGFBP-1 levels compared to those without. Body weight did not appear to affect CXCL13 expression levels. However, long COVID patients with a body mass index (BMI) <18.5 tended to exhibit higher IGFBP-1 levels compared to those with normal weight (18.5 ≤ BMI < 24). The causal relationship underlying this observation remains to be clarified. Taken together, these findings suggest that IGFBP-1 is strongly associated with the presence of long COVID, while CXCL13 may be linked to disease severity.

**TABLE 1:** Serum levels of CXCL13 and IGFBP-1 in long COVID patients across demographic and clinical subgroups^*a*^

Patient characteristic		CXCL13 (pg/mL) median (IQR)	*P* value	IGFBP-1 (ng/mL) median (IQR)	*P* value
Sex	Male (*N* = 31)	12.5 (1.8–160)	0.18	13.8 (1.5–60.2)	0.41
	Female (*N* = 49)	8.7 (0–30.8)		11.6 (2.2–36.9)	
Age	<50 (*N* = 35)	7.8 (0–28.3)	0.24	8.7 (0.6–18.3)	0.0019**
	≥50 (*N* = 45)	10.9 (0.9–94.9)		25.7 (7.1–74.9)	
Severity of long COVID^*b*^	Mild (*N* = 69)	7.8 (0–30.5)	0.0185*	13.0 (2.1–39.1)	0.54
	Moderate to critical (*N* = 11)	44.7 (10.2–252.7)		13.3 (1.0–98.7)	
Comorbidity^*c*^	No (*N* = 45)	4.7 (0–17.4)	0.0014**	9.0 (0.7–27.8)	0.0032**
	Yes (*N* = 35)	26.6 (7.6–117.3)		25.8 (8.7–79.7)	
BMI^*d*^	Normal weight (*N* = 41)	7.6 (0–25.6)	–^*f*^	13.2 (2.0–45.4)	–
	Underweight (*N* = 8)	26.1 (6–415.7)	0.29	51.4 (36.5–109.3)	0.0495*
	Overweight (*N* = 19)	11.5 (0–49)	>0.99	12.96 (1.6–23.2)	>0.99
	Obese (*N* = 12)	8.7 (0–237.2)	>0.99	6.3 (0.3–12.4)	0.46
Smoke^*e*^	Non-smoker (*N* = 63)	10.2 (0–30.2)	–	13.0 (2.2–40.8)	–
	Smoker (*N* = 3)	31 (0–44.7)	>0.99	7.4 (0.26–24.5)	0.78
	Former smoker (*N* = 10)	3.5 (0–164.8)	>0.99	48.9 (8.5–131.1)	0.09

^*a*^ Statistical analyses were performed using the Kruskal-Wallis test and the Mann–Whitney *U* test (*P < 0.05 and **P < 0.01).

^*b*^ The long COVID group was classified into four subgroups based on symptom severity: mild (*N *= 69), moderate (*N *= 6), severe (*N *= 3), and critical (*N *= 2).

^*c*^ Comorbidity refers to the patient's pre-existing conditions, including chronic pulmonary disease, chronic liver disease, chronic kidney disease, hypertension, and diabetes mellitus.

^*d*^ BMI group comparisons were made between the underweight, overweight, and obese groups versus the healthy weight group (underweight BMI <18.5, normal weight 18.5 ≤ BMI < 24, overweight 24 ≤ BMI < 27, obese BMI ≥ 27).

^*e*^ Smoking group comparisons were conducted between non-smokers and smokers and between non-smokers and former smokers.

^*f*^ – indicates that the *P* value is not applicable.

### The antiplatelet drug aspirin prevents the Delta S protein-induced mouse death

**Fig 7:**
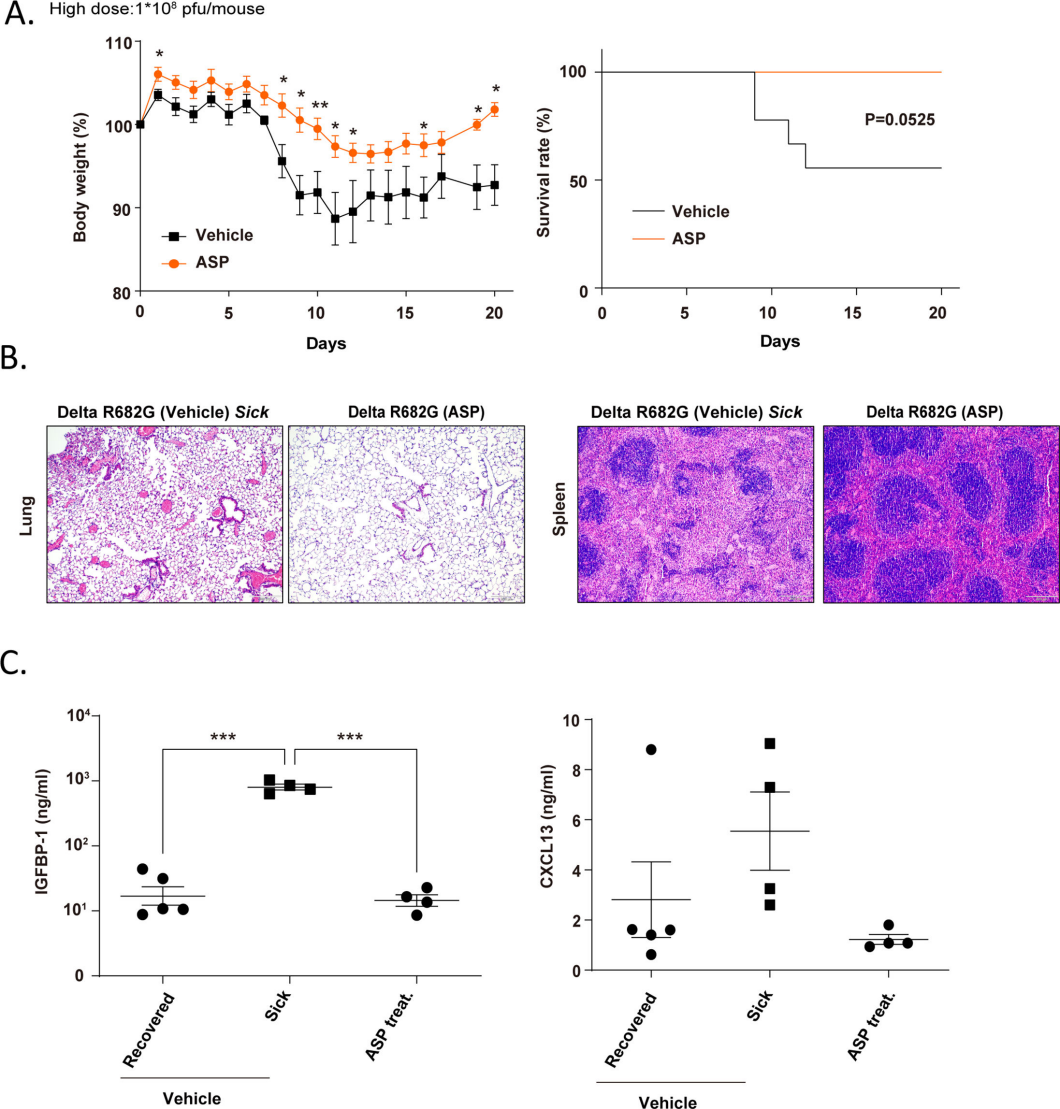
The antiplatelet agent aspirin reduced both mortality and weight loss in Rep-S(Delta R682G) virus-infected mice. (**A**) Transgenic hACE2 mice were intranasally infected with Rep-S(Delta R682G) at a dose of 1 × 10⁸ PFU per mouse. At 24 h post-infection, mice received either aspirin (10 mg/kg; *n* = 7) or vehicle control (*n* = 9) via oral gavage. Treatment was administered once daily for 17 consecutive days (log-rank test and multiple *t-*tests, *P < 0.05, and **P < 0.01). (**B**) Lung and spleen sections were stained with hematoxylin and eosin (H&E). Scale bar: 200 μm. (**C**) Plasma levels of CXCL13 and IGFBP-1 in infected mice were measured by ELISA (one-way ANOVA, ***P < 0.0001).

Since transient expression of the S protein induced delayed pulmonary inflammation, thrombosis, and mortality in mice, the potential of anticoagulation to prevent disease progression was further investigated. Aspirin, an antiplatelet drug with both anticoagulant and anti-inflammatory properties (52–54), was selected for this study. Following Rep-S(Delta R682G) virus infection, mice were treated daily with either aspirin (10 mg/kg) or vehicle control (*n* = 9) via oral gavage. As shown in Fig. 7A, the vehicle-treated group exhibits marked weight loss between days 7 and 10, along with a 45% mortality rate. In contrast, the aspirin-treated group showed significantly less weight loss, gradual recovery beginning around days 15 to 20, and achieved a 100% survival rate. Aspirin treatment reduced lung inflammation and restored splenic tissue integrity (Fig. 7B). It also decreased CXCL13 and IGFBP-1 levels in mice compared to those in vehicle-treated *sick* animals (Fig. 7C). These results suggest that anticoagulation treatment with aspirin can effectively mitigate S protein-induced pathology and prevent mortality.

## DISCUSSION

**Fig 8:**
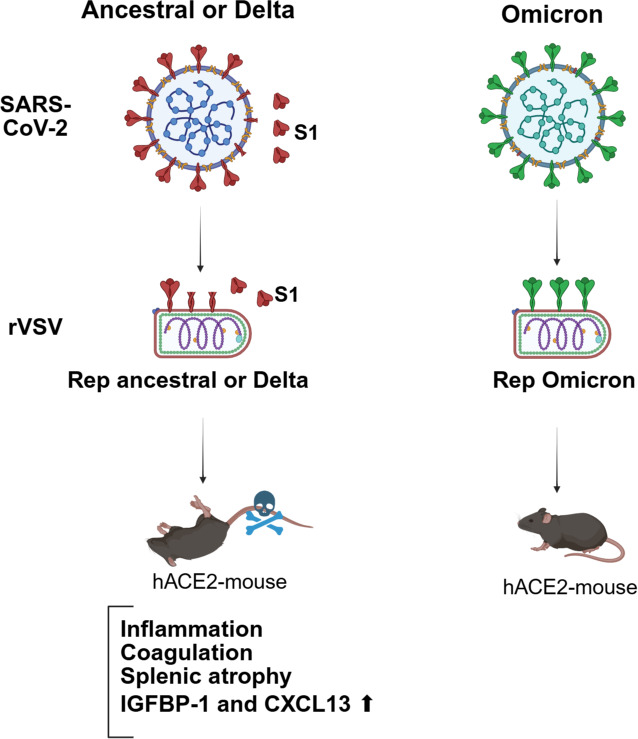
The S protein of SARS-CoV-2 triggers lethal responses in K18-hACE2 mice (created with BioRender.com).

SARS-CoV-2 variants differ in clinical presentation and transmissibility. The Delta variant is associated with more severe disease, whereas the highly transmissible Omicron variant typically causes milder symptoms. These differences appear to be driven, at least in part, by the S protein. The Delta S protein exhibits high cleavage efficiency and leads to extracellular release of S1 subunits. In a mouse model, transient expression of the Ancestral and Delta S protein in mice via a VSV vector leads to gradual coagulation, mild lung inflammation, splenic atrophy, and around 40% mortality within three weeks (Fig. 8). These pathological features and mortality were alleviated by anticoagulant treatment, suggesting that coagulation plays a key role in disease progression. In this model, CXCL13 and IGFBP-1 levels were elevated in the blood and correlated with disease severity. Similarly, in long COVID patients, IGFBP-1 was elevated in serum, and CXCL13 levels were associated with disease severity, reflecting the trends observed in mice. Together, these findings highlight a role for S protein-associated delayed coagulation and inflammation in the development of post-infection sequelae.

After acute SARS-CoV-2 infection, some patients experience long-term symptoms, such as brain fog, fatigue, chest pain, and anxiety (55). In more severe cases, complications can include blood clots, immune dysregulation, autoimmunity, and multi-organ impairment (56–59). These persistent symptoms are collectively referred to as long COVID or post-COVID-19 condition. Thromboinflammation has been observed in active long COVID cases (41, 45), and affected patients face an elevated risk of pulmonary embolism (46). According to self-reported data, approximately 10% of individuals infected with the Delta variant developed long COVID, compared to about 5% of those infected with Omicron (16). Given the similarity between the pathological features observed in mice expressing the S protein and those seen in long COVID patients, this mouse model may serve as a valuable tool for studying the mechanisms underlying long COVID. Because VSV replication is rapidly controlled by the host immune system, expression of the S protein is transient, lasting for only about one day (45). While the VSV vector can induce host immune activation, it has not been linked to coagulation abnormalities (60–63). Thus, the pathological coagulation responses observed in mice exposed to the Ancestral and Delta Rep-S viruses are likely initiated by the S protein on viral particles or by released S1 subunits. Importantly, such effects are not expected after vaccination, as current vaccine platforms do not contain infectious viral particles and do not generate free S1 at levels sufficient to cause pathology.

Several inflammatory molecules, including IGFBP-1 and CXCL13, are elevated in diseased animals following transient expression of the S protein. These molecules may contribute directly to SARS-CoV-2 pathogenesis or serve as potential biomarkers for long COVID. Previous studies have reported elevated CXCL13 levels in patients with severe COVID-19 (64, 65). Although CXCL13 levels in our cohort did not differ significantly between long COVID patients and non-COVID-19 controls, higher levels of CXCL13 were associated with increased disease severity. IGFBP-1, primarily produced in the liver, binds to insulin-like growth factor-1 (IGF-1) and regulates its activity. In deceased COVID-19 patients, elevated levels of IGFBP family members—including IGFBP-1, IGFBP-3, and IGFBP-6—have been reported compared to survivors (66). In our study, IGFBP-1 levels were significantly elevated in long COVID patients compared to individuals without COVID-19 infection. Both CXCL13 and IGFBP-1 appear to play roles in disease progression during the acute phase and in post-infection sequelae. Notably, both markers were elevated in long COVID patients with underlying comorbidities. Whether CXCL13 and IGFBP-1 play direct pathological roles in SARS-CoV-2-associated disease warrants further investigation.

To better contextualize the spike cleavage–related phenotypes observed in our rVSV-based model, the role of the R682G mutation warrants consideration. Our previous work demonstrated that replication-competent rVSV-S (Ancestral) viruses emerged only after serial passaging, during which adaptive mutations—most frequently at residue R682—were acquired to attenuate furin-mediated S1/S2 cleavage and enhance spike incorporation into virions (45). Based on this observation, we directly introduced the R682G mutation into the spike genes of the Delta and Omicron variants to facilitate the rapid generation of replication-competent rVSV-S viruses, thereby eliminating the need for extensive serial passaging. We further compared the expression and processing of Ancestral and Delta spike carrying R682G mutation across multiple experimental systems, including DNA transfection, pseudotyped viruses, and the Rep system. Notably, S proteins in the rVSV–spike system exhibited substantially higher apparent cleavage efficiency than those observed in either the pseudotyped virus or spike expression systems. We speculate that differences in total spike expression levels, virion assembly dynamics, and accessibility to host proteases during rVSV replication likely contribute to the elevated S1 levels detected in rVSV virus particles.

The SARS-CoV-2 Ancestral strain and early variants like Delta predominantly infect the lower respiratory tract, leading to severe complications in COVID-19 patients, such as respiratory failure, pneumonia, coagulation disorders, and hypoxemia. Studies have shown that the S1 subunit contributes to pulmonary injury by increasing vascular permeability, promoting neutrophil recruitment, inducing endothelial cell dysfunction, and activating complement pathways (35, 67, 68). In this study, the Delta variant was found to exhibit enhanced cleavage efficiency at the S1/S2 junction, with detectable secretion of the S1 subunit into the extracellular space. The replication-competent Rep-S(R682G) virus induced coagulation abnormalities and lethality in mice. The additional V705M mutation in Delta S protein may contribute to disease symptoms, and further experiments are required to confirm its role and to delineate the individual contributions of these mutations. In contrast, introducing an additional S813Y mutation into the Delta spike, which disrupts TMPRSS2-mediated cleavage and delays S1/S2 processing, abolished the virus’s ability to induce severe pathology. This suggests that S1 subunit generation and/or release is a key driver of pulmonary coagulation and systemic inflammation. Further investigation is needed to determine the mechanism by which S protein triggers coagulation and immune dysregulation.

## Data Availability

All data supporting the findings of this study are available within the article and its supplemental material.

## SUPPLEMENTAL MATERIAL

Supplemental figures jvi.01255-25-s0001.docx Figures S1 to S9.

Supplemental material jvi.01255-25-s0002.docx Supplemental methods; Tables S1 and S2.

Table S3 jvi.01255-25-s0003.xlsx Cytokine expression in plasma and lung tissue from hACE2 mice infected with VSVdG/G or Rep-S Delta R682G viruses.

ASM does not own the copyrights to Supplemental Material that may be linked to, or accessed through, an article. The authors have granted ASM a non-exclusive, world-wide license to publish the Supplemental Material files. Please contact the corresponding author directly for reuse.
