# Supplementary material for: SARS-CoV-2 spike protein expression drives post-acute coagulopathy
Source: J Virol. 2026 Jan 21;100(2):e01255-25. doi: 10.1128/jvi.01255-25 (PMC12911876; doi:10.1128/jvi.01255-25)
Supplement: Supplemental figures — Figures S1 to S9. [file jvi.01255-25-s0001.docx]

**Figure S1. Delta variant exhibits a higher cleavage efficiency in human alveolar cell.** A549 cells were transfected with plasmid DNA to introduce the expression of spike variants, and spike protein expression in cells was examined by immunoblotting.

**Figure S2. Delta Spike is efficiently transported to the extracellular space.** 293T cells were transfected with plasmid DNA encoding S1(^682^RRAR^685^)-Fc and S1(^682^GSAS^685^)-Fc fusion proteins. The Spike protein expression in cells and culture medium at 36 h post-transfection was examined by immunoblotting. The furin cleavage site (RRAR) is replaced by GSAS sequence to prevent cleavage at the junction between S1 and Fc.

**Figure S2. Delta Spike is efficiently transported to the extracellular space.** 293T cells were transfected with plasmid DNA encoding S1(^682^RRAR^685^)-Fc and S1(^682^GSAS^685^)-Fc fusion proteins. The Spike protein expression in cells and culture medium at 36 h post-transfection was examined by immunoblotting. The furin cleavage site (RRAR) is replaced by GSAS sequence to prevent cleavage at the junction between S1 and Fc.

**Figure S3. SARS‐CoV‐2 S1 induce NET formation.** (A) Murine neutrophils (1 × 10^6^/ml) were incubated with pseudotyped VSV SARS-CoV-2 spike (MOI = 0.5). (B) Murine neutrophils (1 × 10^6^/ml) were incubated with conditional medium collected from HEK293T cells transfected with plasmid DNA encoding spike variants. The supernatant was then used to treat neutrophils. For immunostaining, the neutrophils were stained with anti-myeloperoxidase (MPO) and anti-citrullinated histone H3 (CitH3). Scale bar: 10μm.

**Figure S3. Generation of rVSV-S viruses.** (A) Schematic representation of the genome of rVSV-S variant viruses. Delta and Omicron S gene (wild type, R682G, or R682G+S813Y mutations) with C-terminal 19 amino acid truncation were inserted into VSVΔG-GFP-2.6 plasmid to generate recombinant viruses. N, nucleoprotein; P, phosphoprotein; M, matrix; GFP, green fluorescent protein; L, large polymerase. (B) Additional mutations present in the replication-competent rVSV-S variants are indicated in red.

**Figure S4. Generation of rVSV-S viruses.** (A) Schematic representation of the genome of rVSV-S variant viruses. Delta and Omicron S gene (wild type, R682G, or R682G+S813Y mutations) with C-terminal 19 amino acid truncation were inserted into VSVΔG-GFP-2.6 plasmid to generate recombinant viruses. N, nucleoprotein; P, phosphoprotein; M, matrix; GFP, green fluorescent protein; L, large polymerase. (B) Additional mutations present in the replication-competent rVSV-S variants are indicated in red.

**Figure S5. Comparison of cleavage efficiency of Ancestral and Delta spike with or without the R682G mutation.** (A) 293T cells were transfected with plasmid DNA encoding the indicated Spike variants for 30 hours, and S protein level in cell lysate was examined by immunoblotting. (B) 293T cells were transfected with plasmid encoding S variants for 24 hours followed by infection with VSVΔG/G at a multiplicity of infection (MOI) of 5. Spike-pseudotyped virus particles (Spp) were harvested 24 h post-infection. Cell lysate and Spp were subjected to immunoblotting.

**Figure S6. Ancestral and Delta SARS-CoV-2 variants induce extensive pulmonary fibrinogen deposition compared to Omicron.** Lungs were collected from hamsters infected with live ancestral, Delta, or Omicron strains (1×10^4^ TCID50/mouse) at 6 dpi. Lung sections were subjected to immunohistochemistry with anti-fibrinogen antibody. Scale bar: 200 μm.

**Figure S7. Transient Spike protein expression induces fibrinogen acculmulation in liver.** (A) Liver collected from hACE2-mice infected with VSVΔG/G virus or Rep-S(R682G) (2×10^7^ pfu/mouse) at 20 dpi. Liver sections were stained with H&E. Scale bar: 100 μm. (B) Liver sections collected from hACE2-mice infected with VSVdG/G or Rep-S(R682G) viruses (2×10^7^ pfu/mouse) were subjected to immunohistochemistry with fibrinogen antibody. Scale bar: 100 μm.

**Figure S8. Coagulation related genes are elevated in the Rep-S(Delta R682G) virus-infected mice with symptoms.** Relative mRNA expression levels of (A) F3 (lung), (B) F8 (spleen), and (C) SERPINE1 (liver), collected from Rep-S(R682G) virus-infected mice (2×10^7^ pfu/mouse) were measured by quantitative PT-PCR. The correlation between changes in coagulation factor gene expression and body weight loss at the time of tissue collection was plotted. (Pearson Correlation, **P<0.01)

**Figure S9. The plasma EGF levels from mice infected with VSVdG/G or Rep-S(R682G) viruses (2×10^7^ pfu/mouse) were measured by ELISA.**
