# Supplementary material for: SARS-CoV-2 spike protein expression drives post-acute coagulopathy
Source: J Virol. 2026 Jan 21;100(2):e01255-25. doi: 10.1128/jvi.01255-25 (PMC12911876; doi:10.1128/jvi.01255-25)
Supplement: Supplemental material — Supplemental methods; Tables S1 and S2. [file jvi.01255-25-s0002.docx]

**Supplementary Material and Methods**

**Neutrophil Extracellular Trap Formation and Immunofluorescence Staining**

Bone marrow cells isolated from the femur and tibia of mice were layered onto a discontinuous Percoll gradient (62% and 81%) and centrifuged at 1,500 rpm for 30 min. Neutrophils were collected from the interphase, washed twice with PBS, and resuspended in RPMI medium supplemented with 10% fetal bovine serum (FBS). Murine neutrophils (1 × 10⁶ cells/ml) were cultured on poly-L-lysine–coated coverslips in 12-well plates and exposed to SARS-CoV-2 spike–pseudotyped VSV at a multiplicity of infection (MOI) of 0.5 for 24 h at 37 °C. Dissociated S1-containing conditioned medium was generated by transfecting 293T cells with plasmid DNA encoding different Spike variants. Supernatants were collected 30 h post-transfection and subsequently incubated with murine neutrophils for 24 h at 37 °C. Following incubation with virus or conditioned medium, neutrophils were fixed with 4% paraformaldehyde and permeabilized in PBS containing 1% BSA and 0.03% Triton X-100. Cells were immunostained with antibodies against myeloperoxidase (MPO; R&D Systems, Minneapolis, MN, USA; AF3667) and citrullinated histone H3 (CitH3; Novus Biologicals, Centennial, CO, USA; NB100-57135), followed by nuclear staining with DAPI. Confocal images were obtained using a Leica Stellaris 8 microscope at 630× magnification.

**Fibrinogen deposition in SARS-CoV-2–infected hamster lung tissue**

Hamster lung sections were kindly provided by Dr. Hsin-Wei Chen (National Health Research Institutes, Taiwan). Lung tissues were collected from female Golden Syrian hamsters (15–18 weeks old) infected intranasally with 1 × 10⁴ TCID₅₀ of SARS-CoV-2 Ancestral (hCoV-19/Taiwan/4/2020), Delta (hCoV-19/Taiwan/1144/2021), or Omicron (hCoV-19/Taiwan/16804/2021) strains at 6 days post-infection. The tissue sections were subjected to immunohistochemistry using an anti-fibrinogen antibody (Dako; Agilent).

**Quantitative Real-Time PCR**

RNA was extracted from murine organ homogenates using TRIzol LS (ThermoFisher), and subsequently treated with DNase I (Thermo scientific) to remove chromosomal DNA. The cDNA was synthesized with FIREScript® RT cDNA synthesis KIT (Solis BioDyne, Tartu, Estonia). Expression levels of coagulation-related genes (F3, F8, and SerpinE1) in the synthesized cDNA were detected by Real-time quantitative-polymerase chain reaction (qPCR) using specific primers and SYBR green dye (Luminaris Color HiGreen qPCR Master Mix, ThermoFisher) in an Applied Biosystems QuantStudio 6 Flex real-time PCR system. Quantification cycle (Cq) of each gene was determined and normalized with cyclophilin A (Cph) housekeeping gene expression level. Relative gene expression level was calculated using the ΔΔCq method. The gene-specific primer sequences are listed in Supplemental Table. S1.

### Sequence-independent, single-primer amplification (SISPA) & Nanopore sequencing

### RNA was extracted from murine lung homogenates using QIAzol Lysis Reagent (QIAGEN, Cat#79306), while DNA extraction from murine lung homogenates was performed using the HostZERO™ Microbial DNA Kit (ZYMO RESEARCH, Irvine, CA, USA). The extracted RNA, serving as a template, was transcribed into complementary DNA (cDNA) using SuperScript IV Reverse Transcriptase (ThermoFisher, Cat #18091200) and random primers. Subsequently, the cDNA was amplified with Sequenase (ThermoFisher, Cat#70775Y200UN). In contrast, the DNA was denatured at 95°C for 10 mins. The denatured DNA was then annealed with random primers at 65°C for 5 mins and amplified with Sequenase (ThermoFisher). The second round of amplification for both RNA and DNA was carried out using AccuTaq LA DNA Polymerase (SigmaAldrich, Cat#D8045-125UN) and tagged sequence random primers. The microbial DNA and RNA sequences were analyzed using Nanopore (GridION, sequencing), NanoLyse (remove host genome), and Centrifuge software (sequence alignment with virus and bacterial).

**Tables**

Table S1. Primers for Real-time quantitative-polymerase chain reaction

| Gene |  | Sequence (5’->3’) |
| --- | --- | --- |
| F3 | F | AACCCACCAACTATACCTACACT |
|  | R | GTCTGTGAGGTCGCACTCG |
| F8 | F | CTTCACCTCCAGGGAAGGACTA |
|  | R | TCCACTTGCAACCATTGTTTTG |
| SERPINE1 | F | CGACAGCCAACAAGAGCCAATC |
|  | R | AACCCTTTCCCAGAGACCAGAACC |
| CPH | F | ATGGTCAACCCCACCGTGT |
|  | R | TTCTTGCTGTCTTTGGAACTTTGTC |

Table S2. Serum GOT and GPT levels in mice.

|  | Mock  (pre-serum) | Serum from  Delta R682G-exposed sick mice | | | | |
| --- | --- | --- | --- | --- | --- | --- |
| GOT  (U/L) | 42 | 505 | 682 | 469 | 495 | 611 |
| GPT  (U/L) | 24 | 165 | 92 | 160 | 73 | 215 |
